# Chromosome Territory Modeller and Viewer

**DOI:** 10.1371/journal.pone.0160303

**Published:** 2016-08-09

**Authors:** Magdalena A. Tkacz, Kornel Chromiński, Dominika Idziak-Helmcke, Ewa Robaszkiewicz, Robert Hasterok

**Affiliations:** 1 Institute of Computer Science, Faculty of Material and Computer Science, University of Silesia in Katowice, Sosnowiec, Poland; 2 Institute of Technology and Mechatronics, Faculty of Material and Computer Science, University of Silesia in Katowice, Sosnowiec, Poland; 3 Department of Plant Anatomy and Cytology, Faculty of Biology and Environmental Protection, University of Silesia in Katowice, Katowice, Poland; Julius Kuehn-Institute (JKI), GERMANY

## Abstract

This paper presents ChroTeMo, a tool for chromosome territory modelling, accompanied by ChroTeVi–a chromosome territory visualisation software that uses the data obtained by ChroTeMo. These tools have been developed in order to complement the molecular cytogenetic research of interphase nucleus structure in a model grass *Brachypodium distachyon*. Although the modelling tool has been initially created for one particular species, it has universal application. The proposed version of ChroTeMo allows for generating a model of chromosome territory distribution in any given plant or animal species after setting the initial, species-specific parameters. ChroTeMo has been developed as a fully probabilistic modeller. Due to this feature, the comparison between the experimental data on the structure of a nucleus and the results obtained from ChroTeMo can indicate whether the distribution of chromosomes inside a nucleus is also fully probabilistic or is subjected to certain non-random patterns. The presented tools have been written in Python, so they are multiplatform, portable and easy to read. Moreover, if necessary they can be further developed by users writing their portions of code. The source code, documentation, and wiki, as well as the issue tracker and the list of related articles that use ChroTeMo and ChroTeVi, are accessible in a public repository at Github under GPL 3.0 license.

## Introduction

Almost every living eukaryotic cell contains genetic material in the form of chromatin consisting of linear DNA molecules and DNA-associated proteins, enclosed inside the cell nucleus. During a cell division, chromatin fibres condense into rod-like structures called chromosomes, which enables balanced and efficient segregation of genetic material into two daughter cells. During the period between two subsequent divisions, called interphase, chromosomes decondense and occupy distinct 3-D areas within the nucleus, known as chromosome territories (CTs) [1]. Chromosome territories can be experimentally identified using cytomolecular approach of *in situ* hybridisation with fluorescently labelled DNA probes (FISH) that enables specific discrimination and visualisation of individual chromosomes (so-called chromosome painting; CP) [2, 3]. This methodology is now routinely applied in many animal species, primarily mammals and birds providing useful data about the relationship between the internal architecture of the cell nucleus and such crucial intranuclear processes as regulation of gene expression, gene transcription or DNA repair [4–6]. In plants, similar studies are scarce due to the difficulties in obtaining chromosome-specific painting probes which is caused by vast amounts of repetitive DNA sequences dispersed throughout the genomes of most plant species. So far the most complex data about the CT distribution within the plant nucleus were obtained for a model dicotyledonous plant *Arabidopsis thaliana*. They revealed that the arrangement of CTs in this species was predominantly random except for the chromosomes bearing the nucleolar organising region [7]. Recently, similar opportunity to study nucleus structure appeared for another plant species, monocotyledonous *Brachypodium distachyon*, which is a model grass for the temperate zone cereals such as barley, wheat and rye [8, 9]. In order to assess whether CTs in *B*. *distachyon* are distributed randomly or are subjected to certain patterns, the experimental data have to be complemented by a computer simulation. The model proposed in this work reflects the decondensation process of the post-mitotic chromosomes within the given sphere until the entire space is uniformly filled with simulated chromatin fibres. Decondensed chromatin is rendered as a chain of 1 Mbp domains (500 nm in diameter), which are supposed to be the basic structural units building up CTs [10, 11]. The simulation also takes into account the number of chromosomes and their morphological features such as total length and the position of the centromere. It also enables to discriminate between two homologous chromosomes and between two chromosome arms. As the nuclei in the living cells vary in terms of their size and shape as well as the size and position of their nucleoli, the optimal model should consider and allow to adjust these parameters.

## Design and Implementation

### Model parameters

The model presented in this work ensures fully probabilistic distribution of CTs in the interphase nucleus. The aim of such approach was to establish if the position of CTs in the nuclei of *B*. *distachyon* is random or if some chromosomes associate more or less often. The comparison between frequencies of associations of chromosome/chromosome arm territories obtained through the simulation and the experiments should enable to answer this question. While developing the model, the parameters determining visualisation were adjusted to facilitate the visual comparison with photomicrographs demonstrating the experimental data obtained by FISH-based CP.

**Fig 1:**
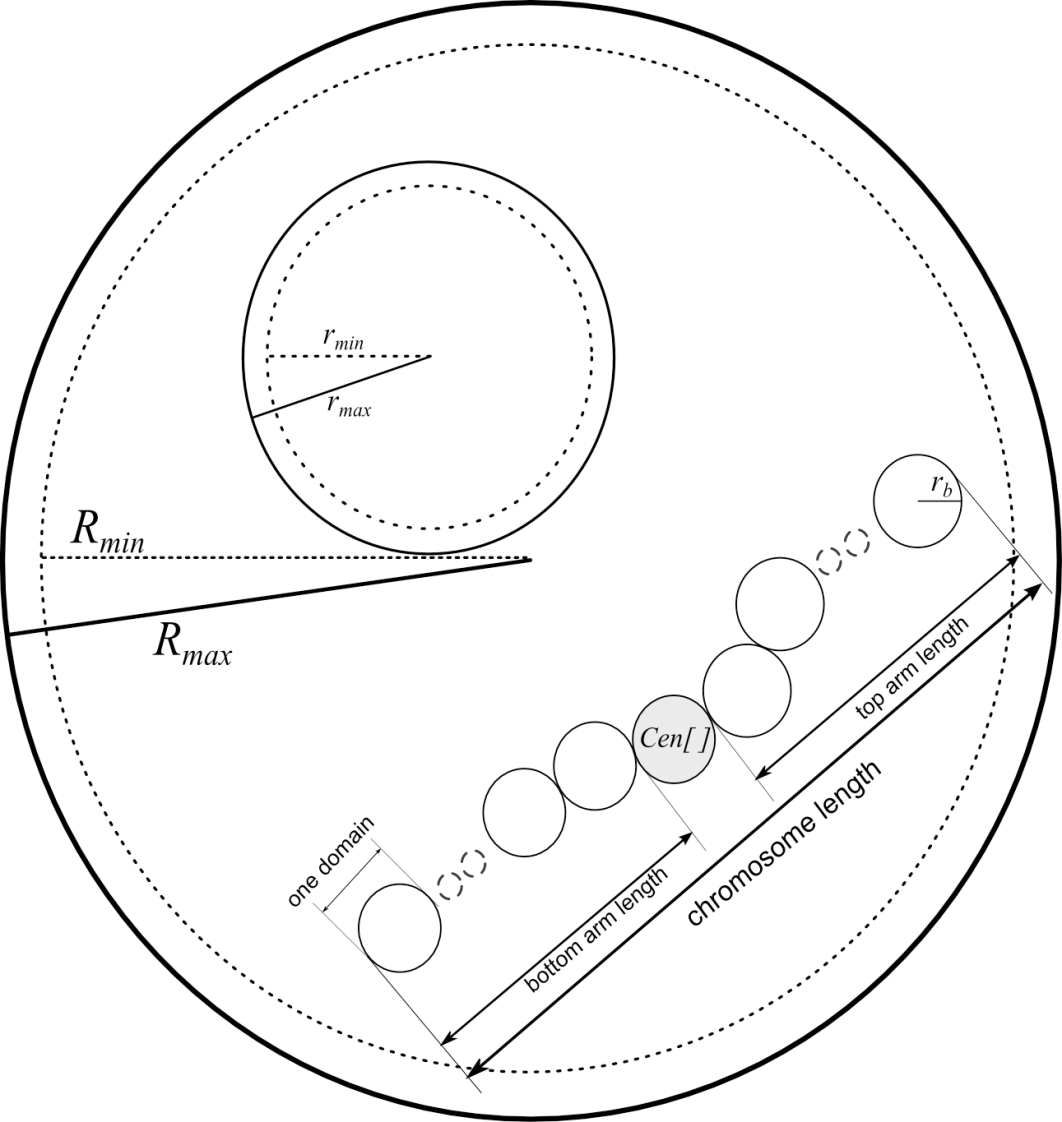
Model parameters and their visual presentation. Nucleus has radius *R* that varies in range from *R*_*min*_ to *R*_*max*_. Nucleolus has radius *r* that varies in range from *r*_*min*_ to *r*_*max*_. The chromosome in its condensed state is represented by a chain of spherical domains with fixed radius *r*_*b*_. Each chromosome has one centromere *Cen[]* and consists of a top and bottom arm with a given length.

Among the assumed model parameters, *R* is a radius of the nucleus (Fig 1) that varies in range from *R*_*min*_ to *R*_*max*_ (*R ∈* (*R*_*min*_, *R*_*max*_)), while *r* stands for a radius of the nucleolus and varies in range from *r*_*min*_ to *r*_*max*_ (*r ∈* (*r*_*min*_, *r*_*max*_)). The number of chromosomes in the nucleus is given by *N*. Each chromosome has one centromere *Cen[N]* and consists of two arms with a given length (Fig 1). The length of arms is represented as two parameters: first when in condensed form and second–after decondensation.

In our model, one 1 Mbp domain is regarded as one bead, which is visualised as a sphere with fixed radius *r*_*b*_ and with the centre in the three-dimensional space. The radius of spheres in the model should be chosen from range 400 *−* 800 nm according to the data found in the literature [10, 11]. This parameter should be set carefully since too high value will result in sub-optimal space filling in the nucleus, whereas too low value will cause longer running time and more complicated shapes.

### Steps of modelling process

**Fig 2:**
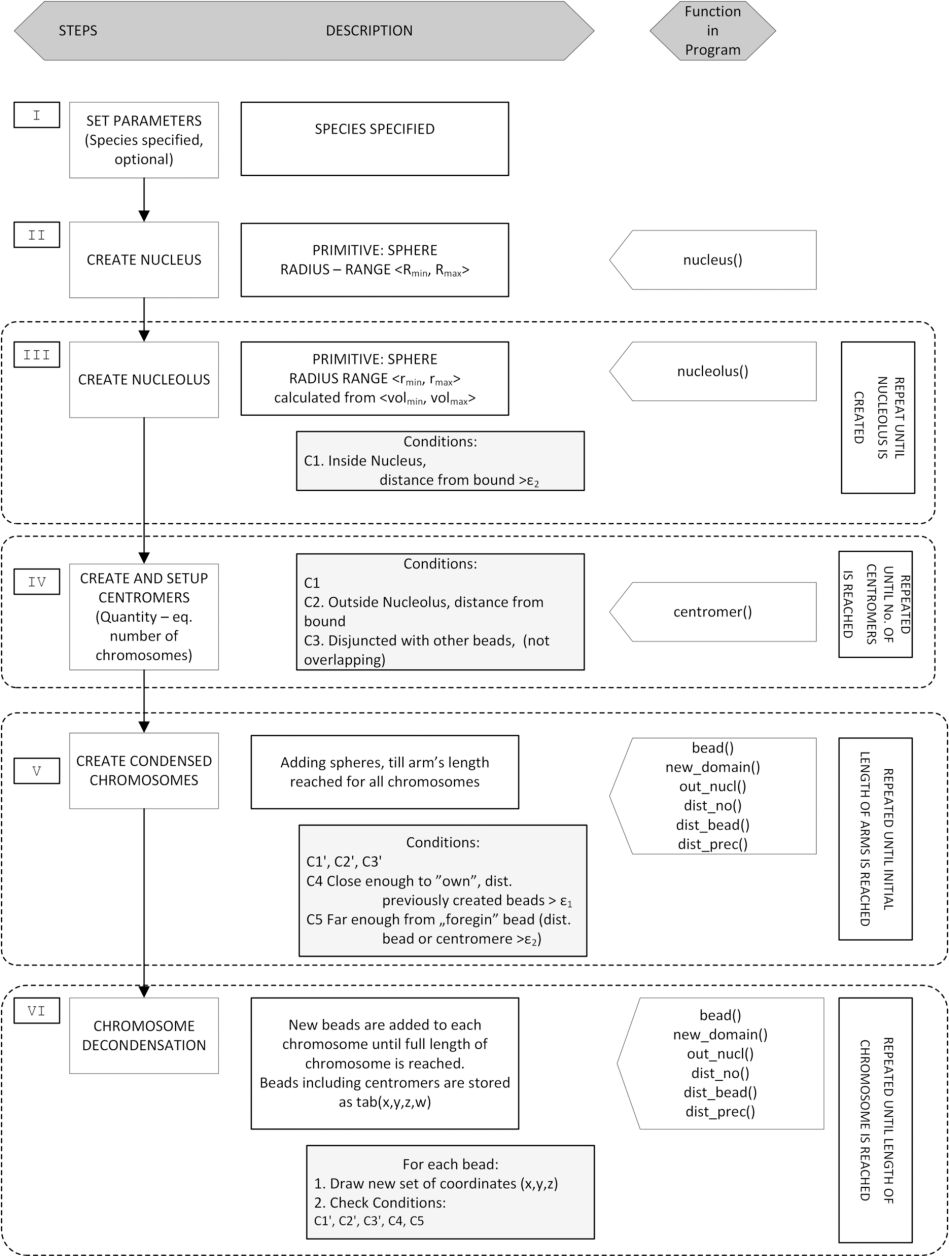
Steps of modelling. The modelling process is divided into six blocks (numbers I–VI). The middle column gives more detailed description of each step, including the conditions (C1-C5 and C1’-C3’) that have to be met for the program to proceed.

The process of CTs modelling can be divided into several steps (Fig 2):

Setting up initial parameters for the model;

Creating nucleus;

Creating nucleolus;

Setting up position and creating centromeres;

Creating chromosomes in condensed state;

Simulating chromatin decondensation process.

Each step is followed by checking against the collision of the newly created components with already existing ones. This is ensured by checking several conditions and is later referred to as so-called collision detection procedure.

In the beginning, setting up some parameters is necessary. These include species–specific features, such as the number and length of the chromosomes, as well as the position of the centromeres. This step is represented by block I in Fig 2. In the second step, a nucleus is created. The nucleus is drawn as a sphere. Its size may vary and is species specific. Therefore, it should be determined basing on experimental observations. The centre of the nucleus is the centre of the canvas and has coordinates *nu* = (*x*_*nu*_, *y*_*nu*_, *z*_*nu*_) = (0, 0, 0). This step is represented as block II in Fig 2. No additional conditions are necessary in this stage.

**Fig 3:**
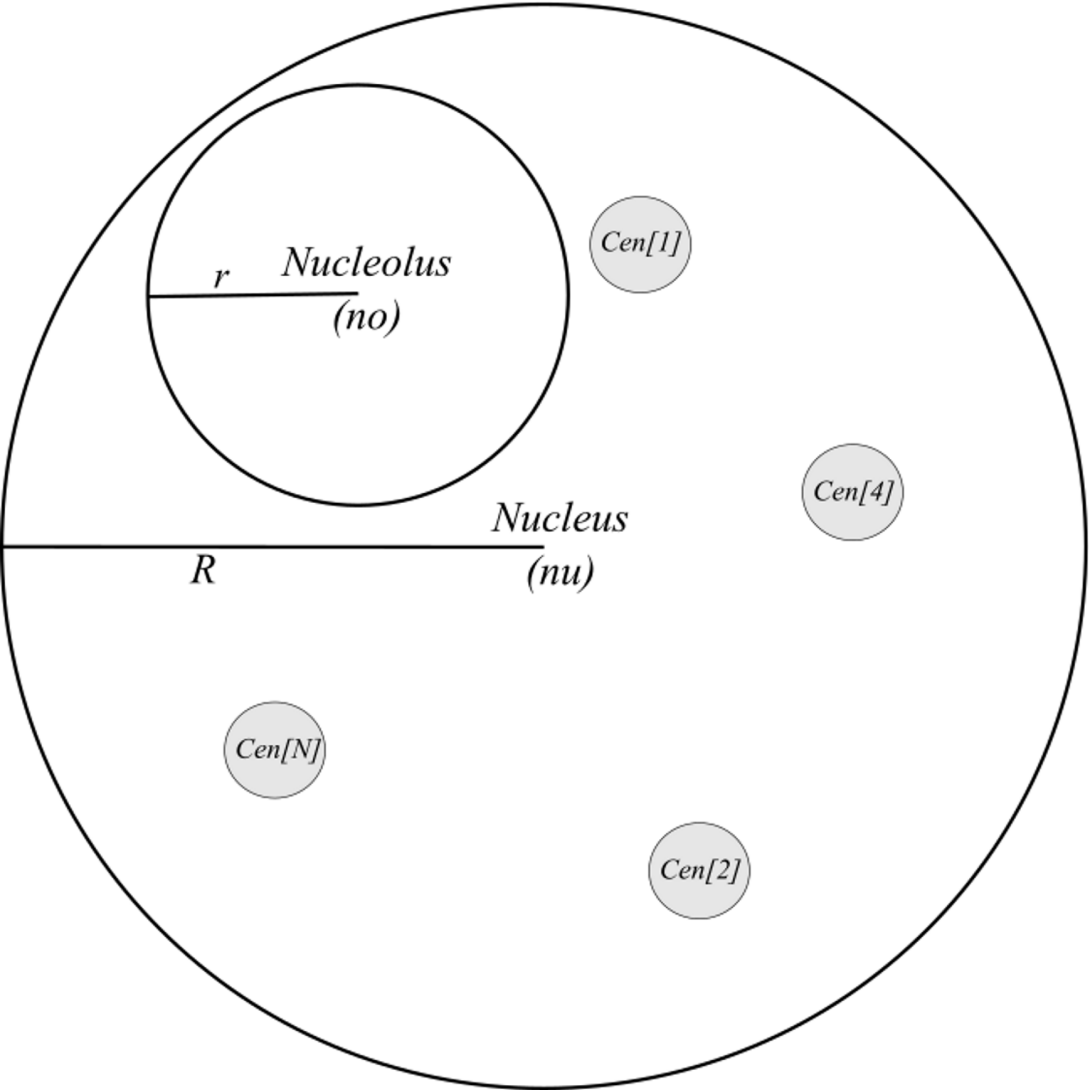
State of the model after I-IV steps of the modelling process. The spheres representing nucleus, nucleolus and the centromeres of *N* chromosomes are drawn. *R* and *r* stand for the radius of the nucleus and nucleolus, respectively while (*nu*) and (*no*) stand for the coordinates of their centres, also respectively. Checking the conditions **C1–C3** (see text) ensures that the nucleolus and the centromeres are located inside the nucleus and that the drawn structures do not collide with each other.

The third step is nucleolus creation (block III in Fig 2, Fig 3). The radius of a sphere representing the nucleolus is chosen from a range (*r*_*min*_, *r*_*max*_) and computed using the input parameters *vol*_*min*_ and *vol*_*max*_ according to the Eq (1). The parameters *vol*_*min*_ and *vol*_*max*_ should be determined through experiments.

In this step the first condition, **C1** appears:

- **C1** –the nucleolus has to be inside the nucleus and not overlap with the nucleus boundary. This is ensured by setting up the coordinates *no* = (*x*_*no*_, *y*_*no*_, *z*_*no*_) of the centre of the nucleolus according to the Eq (2).

During the fourth step (block IV in Fig 2, Fig 3), centromere positions are set up and the spheres representing centromeres are drawn. The number of chromosomes *N* and the length of chromosome arms can be set as species-specific model parameters.

In this step, the drawn coordinates must meet three conditions:

**C1 –**the sphere representing a centromere has to be inside the nucleus and cannot overlap with the nucleus boundary (see Eq (3)),
**C2 –**the coordinates of the centre of the sphere that represents a centromere have to be located outside nucleolus (see Eq (4));
**C3 –**the coordinates of the centre of the sphere that represents a centromere cannot collide with other centromeres (no other centromere can be present within distance of 2*· r*_*b*_ + *ε*_2_, see Eq (5)).

**Fig 4:**
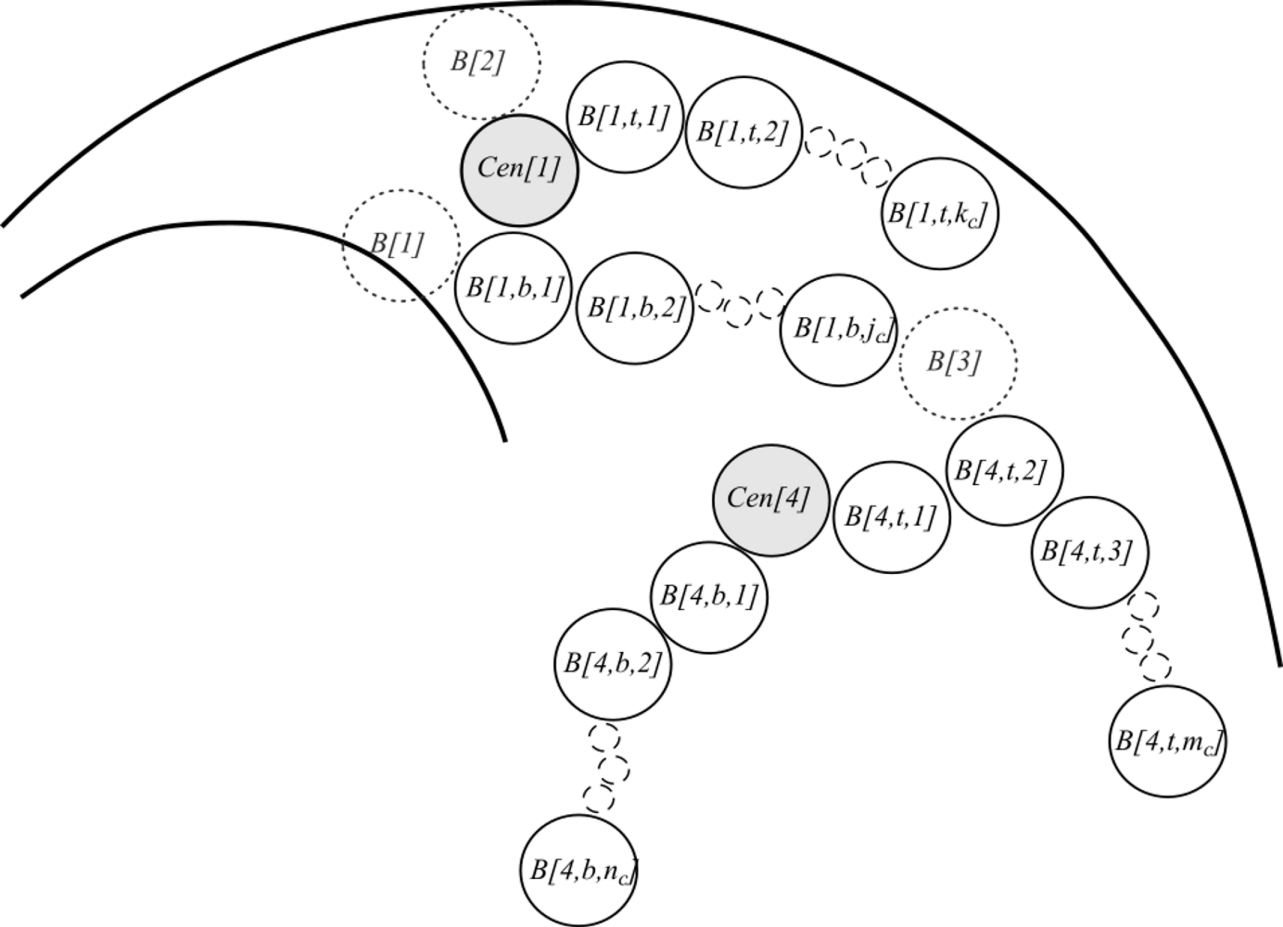
Step V of the modelling process: creating condensed chromosomes. Already existing beads (solid line circles) are named by assigning them a letter *B* followed by three numbers: identifier of the chromosome, identifier of the chromosome arm (*t*–top or *b*—bottom), and identifier of a bead in each arm. For example, *B(4*,*t*,*5)* should be read as bead number 5, in top arm of chromosome 4. Temporary beads (dotted line circles), which are introduced only for presentation purposes are numbered with the use of only one identifier which is a single number. The temporary beads (”bead candidates”) *B1*, *B2*, and *B3* will be discarded because they do not pass”collision detection” procedure, i.e. *B1* collides with the nucleolus (condition **C2** is not met), bead *B2* collides with the nucleus boundary (condition **C1** is not met) and *B3* (which belongs to chromosome 4) is too close to *B[1*, *b*, *j*_*c*_*]* and does not meet the condition **C3**.

After setting the initial components, the coordinates of subsequent beads are drawn to create entire chromosomes in condensed state (block V in Fig 2, Fig 4). To accomplish this, the new beads are added bidirectionally, starting from the centromeres until they reach the length of arms of a given chromosome in condensed state. During this step, each set of the coordinates of a potential bead is checked according to the following conditions:

**C1’**, **C2’**, **C3’–**with similar function to **C1**, **C2**, **C3** mentioned above, but according to the Eqs: (7), (8), (10) accordingly;

condition **C4 –**coordinates should be”close enough” to the”own” chromosome (all beads of a chromosome, see Eq (9));

condition **C5 –**coordinates should be”far enough” from a”foreign” chromosome. This condition is applied to all beads of each chromosome, see Eq (11).

**Fig 5:**
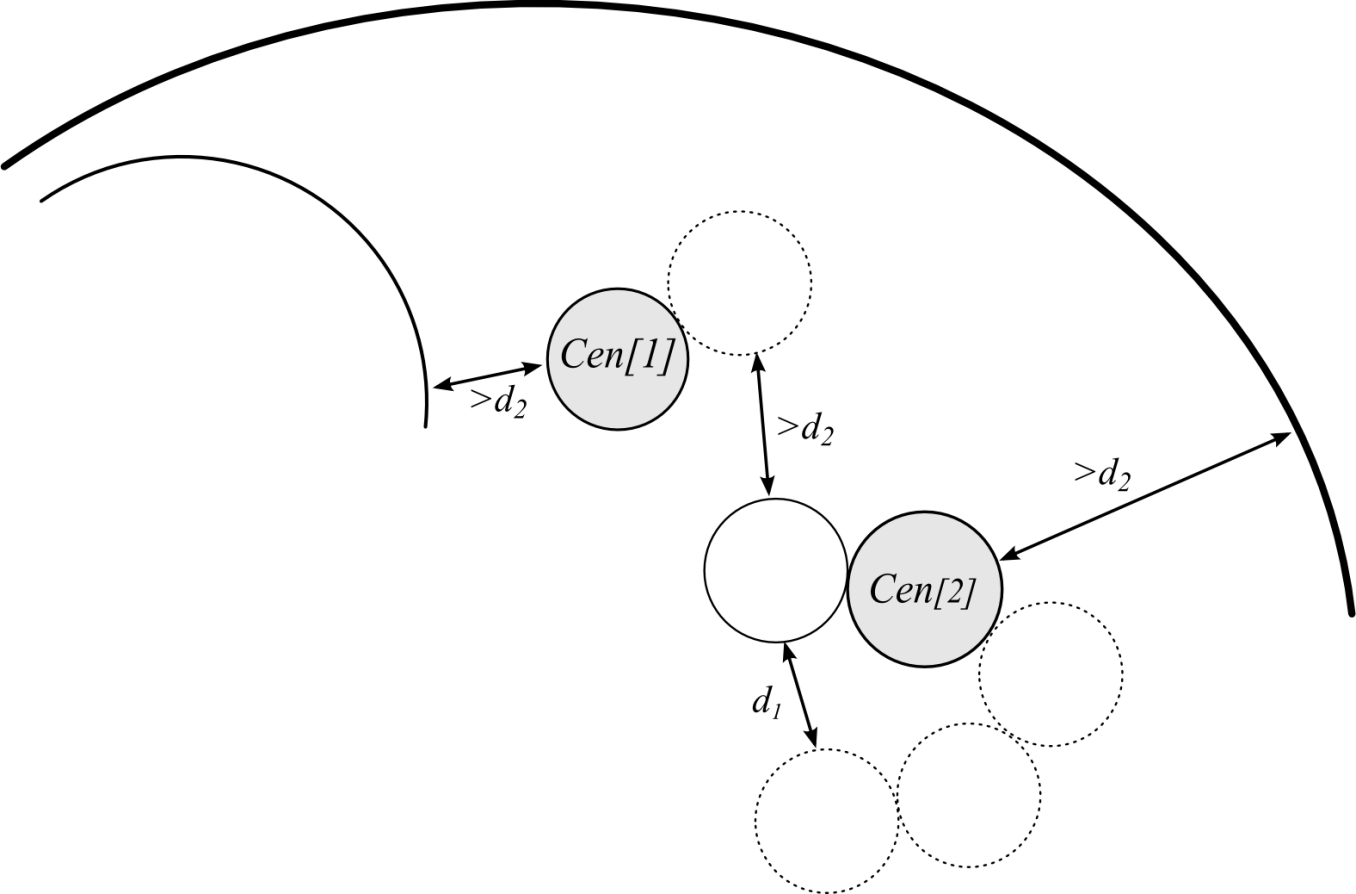
The distances between the beads and other nucleus components. The distance between the beads belonging to the same chromosome is shown as *d*_*1*_ and is calculated using parameter *ε*_*1*_. Two subsequently drawn beads are tangent. The distance between the beads belonging to different chromosomes or between the beads and nucleus or nucleolus boundary is shown as *d*_*2*_ and is calculated using parameter *ε*_*2*_. *Cen[1]* and Cen *[2]* represent centromeric beads (grey circles).

We use two distances *d*_*1*_ (1) and *d*_*2*_ (2) to check the distance between the components (Fig 5):

$$2\cdot r_{b}\leq d_{1}\leq R-2\cdot r_{b}-\varepsilon_{1}$$ (1)

when the distance is measured to”own” beads, and:

$$\varepsilon_{2}\leq d_{2}\leq R-r_{b}-5\cdot \varepsilon_{2}$$ (2)

when the distance is measured to”foreign” beads and the boundaries of nucleus and nucleolus.

Values of *ε*_1_ and *ε*_2_ has been set up experimentally and can be changed inside the code.

**Fig 6:**
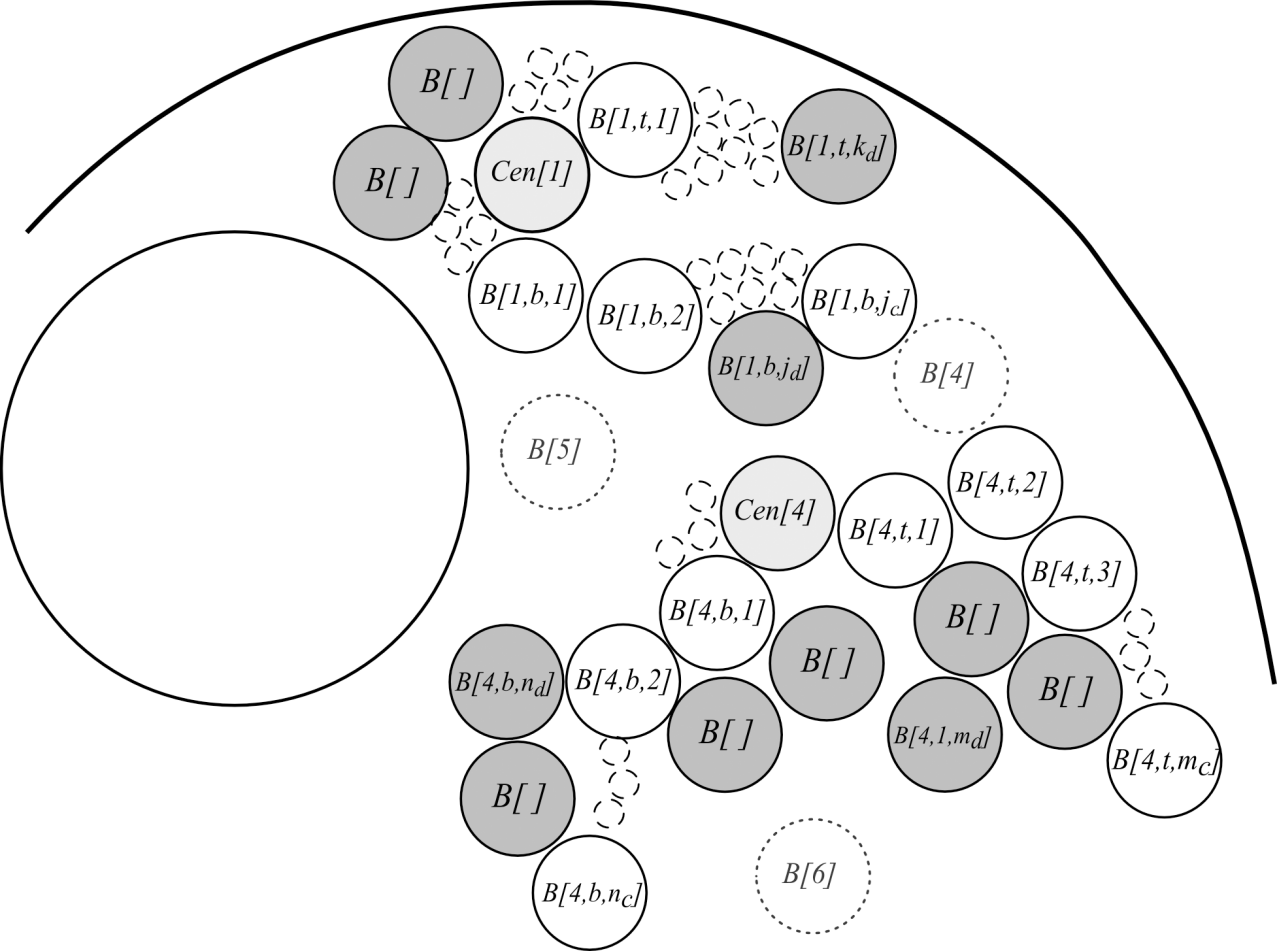
Step VI of the modelling process: simulating chromatin decondensation. The condensed state of a chromosome is represented by a chain of beads identified with three numbers (white circles). The chromosome decondensation is simulated by adding new beads (grey circles) along the length of the entire chromosome, not only at the last created bead. The coordinates of the new beads are generated randomly. The candidate beads *B4*, *B5*, *B6* (dotted line circles) will be discarded because they do not pass collision detection procedures: *B4* and *B5* are too far from the”own” chromosome (condition **C4** is not met), *B6* is too close to the”foreign” beads (condition **C5** is not met).

When the initial structure of the nucleus is created, the simulation proceeds to the step VI of modelling process (see block VI in Fig 2), which is simulating of chromatin decondensation. In this step, the coordinates for the new beads are drawn until the length of each arm (equal to the number of 1 Mbp domains for each arm) reaches the value that was set up in initial parameters. The new beads are added along the length of the entire chromosome, not only at the last created bead. This is done by subsequently drawing a set of coordinates for a new bead (for each chromosome,”around” each chromosome) and checking if all previously defined conditions **C1’**–**C3’**, **C4**, and **C5** are met. Should even one condition fail, drawn coordinates are discarded and a new set is drawn and checked against collision with the existing components of nucleus (see Fig 6).

### Implementation details

The package consists of two scripts. The first one, ChroTeMo (**Chro**mosome **Te**rritory **Mo**deller) is meant to create CT arrangement. This script is responsible for creating a model. It also enables to view and analyse CTs, and the results can be saved to a text file. The second script, ChroTeVi (**Chro**mosome **Te**rritory **Vi**ewer) allows to view and analyse results from ChroTeMo generated files.

Both ChroTeMo and ChroTeVi are implemented in Python. Python [12] is a high-level, object-oriented, general purpose programming language, easy to use and read, and convenient for fast prototyping [13, 14]. It is also portable (multiplatform), very extensible and modular. From the wide selection of extensions available for Python, we chose VPython to make the implementation. With VPython library we were able to make simulation and visualisation. We used VPython together with VIDLE (Visual Integrated Development Environment) which is a part of VPython package.

In VPython, the centre of canvas (window—scene where a picture is to be painted) has (0, 0, 0) coordinates [13]. Entire scene can be zoomed in, zoomed out, and rotated with the use of the mouse and mouse wheel. VPython has predefined 3-D objects named primitives. Complete list of primitives is available in VPython documentation [14, 15].

For the purpose of creating our model and visualisation, we used one of the primitives–a sphere. The sphere is defined by setting up coordinates of its centre *x*_*s*_, *y*_*s*_, *z*_*s*_ and radius. Our implementation was made and tested on Python 2.7, numpy [15] version 1.6.2 and VPython v.6.05. Minimal requirements to run the program are:

OS: Windows (XP or higher), Linux, Mac;

Processor: Pentium 4 (1 GHz or higher);

RAM: 512 MB (1 GB preferred).

The repository for this project is located at Github (https://github.com/Kornelch/ChroTeMoVi). Our code is released under the GPLv3 [16] license. The list of accompanying articles that utilise ChroTe suite can be found in the ‘Satellite Articles’ file in the repository. This list will expand as the other related papers and application studies are successively added.

#### Model variables in ChroTeMo

In our model there are some parameters, which can help to tailor and tune up the code for the simulation of chromosome territory arrangement in different species. When fitting implementation for a given purpose the following parameters can be set up:

chr_pair–number of chromosome pairs to be generated;
l_arm_c–table of length of the arms of a condensed chromosome;
l_arm_d–table of length of the arms of a decondensed chromosome;
min_rad_nu–*R*_*min*_, the minimum radius of the nucleus;
max_rad_nu–*R*_*max*_, the maximum radius of the nucleus;
min_vol_no–*vol*_*min*_, minimum volume of the nucleolus (expressed as percentage of nucleus volume);
max_vol_no–*vol*_*max*_, maximum volume of the nucleolus (expressed as percentage of nucleus volume);
rad_bead–*r*_*b*_, radius of a chromatin domain;
eps_1 –*ɛ*_*1*_, a parameter used in collision detection procedure: minimum distance from another domain of the same chromosome;
eps_2 –*ε*_2_, a parameter used in collision detection procedure: minimum distance from another domain of a different chromosome, also minimum distance from the boundaries of the nucleus and nucleolus;
multi–multiplication: allows to increase the number of beads constituting one domain representation (*1 domain = multi · 1 bead = multi · 1 sphere*). In the presented version of ChroTeMo, one domain is represented by one bead (*multi* = 1) and these two terms are used interchangeably in the latter parts of the text.
restart_after–the value of this parameter indicates the number of iterations before the modeller, being unable to add a new domain to the previously created model components, restarts.

#### ChroTeMo functions

**nucleus()** The purpose of this function is to create the nucleus. The nucleus is represented by a Python primitive–sphere, with the coordinates of the centre (x_nu_, y_nu_, z_nu_) = (0, 0, 0), and with the radius *R* generated in a random way from the interval < *R*_*min*_, *R*_*max*_ >.

**nucleolus()** This function is used to generate the nucleolus. In the first step the radius of nucleolus *r* is determined using parameters *vol*_*min*_ and *vol*_*max*_, according to (3).

$$\begin{array}{l} r_{\min}=\sqrt[{3}]{\left(vol_{\min}\cdot R^{3}\right)}\\ r_{\max}=\sqrt[{3}]{\left(vol_{\max})\cdot R^{3}\right)}\\ r=random.uniform(r_{\min},r_{\max}) \end{array}$$ (3)

In the next step the coordinates of the nucleolus *x*_*no*_, *y*_*no*_, *z*_*no*_ are generated. Since the entire nucleolus has to be inside the nucleus, coordinates must comply the condition III-C1 (Fig 2) which is calculated according to the Eq (4).

$$\begin{array}{l} x_{no}=random.uniform((x_{nu}-(R-r-1)),(x_{nu}+(R-r-2)))\\ y_{no}=random.uniform((y_{nu}-(R-r-1)),(y_{nu}+(R-r-2)))\\ z_{no}=random.uniform((z_{nu}-(R-r-1)),(z_{nu}+(R-r-2))) \end{array}$$ (4)

**centromere()** This function is responsible for generating the coordinates of the centromere bead centres for all chromosomes. The coordinates of the centromeres are stored in an array xyz[]. Here the verification whether the coordinates are inside nucleus and outside of the nucleolus is performed, as well as the collision detection with previously created centromeres. In the formulas below, *i* is an index of a centromere which is presently generated and which coordinates have to be verified.

To test if the coordinates fall into the interior of the nucleus, Eq (5) is used.

$$\sqrt{\left({\left(xyz[0][i]-x_{nu}\right)}^{2}+{\left(xyz[1][i]-y_{nu}\right)}^{2}+{\left(xyz[2][i]-z_{nu}\right)}^{2}\right)}<(R-(r_{b}+5\cdot \varepsilon_{2}))$$ (5)

The formula (6) is used in order to check against the collision with the nucleolus.

$$\sqrt{\left({\left(xyz[0][i]-x_{no}\right)}^{2}+{\left(xyz[1][i]-y_{no}\right)}^{2}+{\left(xyz[2][i]-z_{no}\right)}^{2}\right)}>(r+r_{b}+\varepsilon_{2})$$ (6)

To check the possibility of the collision with another centromere, Eq (7) is used, where *j* is the index of the other chromosome.

$$\sqrt{\left({\left(xyz[0][i]-xyz[0][j]\right)}^{2}+{\left(xyz[1][i]-xyz[1][j]\right)}^{2}+{\left(xyz[2][i]-xyz[2][j]\right)}^{2}\right)}>(2\cdot r_{b}+\varepsilon_{2})$$ (7)

If newly drawn coordinates for possible centromere position meet all the conditions described above, their values, together with the index of the chromosome, are written to the arrays tab[], which stores the coordinates of all beads, and xyz[], which is a temporary array and stores only the coordinates of the last generated beads.

**bead()** This function is used to draw all new domains of all chromosomes. The input parameters for this function are domain coordinates, colour (RGB colour defined as a separate degree of saturation for each component), and transparency. At the initial stage of the modelling process this function is also responsible for drawing centromeres as spheres.

**new_domain()** This function generates the coordinates of the new domain. For domains that are drawn during building the condensed chromosomes, the new coordinates are generated based on last generated coordinates (within the domain of the same arm). For the domains that are drawn in the phase of chromatin decondensation, the coordinates of the next domain are determined on the basis of a randomly selected domain (referred to as a precursory domain) from all previously generated domains that constitute chromosome arms. In this function, the transformation between Cartesian and spherical coordinates is performed according to [17]. Coordinates are generated according to the Eq (8).

$$\begin{array}{l} \theta =random(0,\Pi )\\ \phi =random(0,2\cdot \Pi )\\ xyz[0][n_{b}]=xyz[i][n_{b}]+r_{b}\cdot \cos(\theta )\cdot \sin(\phi )\\ xyz[1][n_{b}]=xyz[i][n_{b}]+r_{b}\cdot \sin(\theta )\cdot \sin(\phi )\\ xyz[2][n_{b}]=xyz[i][n_{b}]+r_{b}\cdot \cos(\phi ) \end{array}$$ (8)

where *n*_*b*_ is the index of the presently processed bead.

**is_in_nu()** This function is used to check whether the new domain is inside the nucleus. In order to detect the collision with the nucleus boundary, the Eq (9) is used.

$$(R-r_{b})>\sqrt{\left({\left(xyz[0][n_{b}]-x_{nu}\right)}^{2}+{\left(xyz[1][n_{b}]-y_{nu}\right)}^{2}+{\left(xyz[2][n_{b}]-z_{nu}\right)}^{2}\right)}$$ (9)

If the condition is true, it means that the newly generated domains would not get into the interior of the nucleus, and the function is invoked once again to generate a new set of coordinates for a new domain.

**is_out_no()** This function is used to detect if the coordinates generated for the new bead are outside the nucleolus according to the Eq (10).

$$\sqrt{({\left(xyz[0][n_{b}]-x_{no}\right)}^{2}+{\left(xyz[1][n_{b}]-y_{no}\right)}^{2}+{\left(xyz[2][n_{b}]-z_{no}\right)}^{2}}<(r+r_{b}+\varepsilon_{2})$$ (10)

If the above condition is true, the program proceeds to draw new coordinates for the next domain.

**dist_bead()** This function is used to check whether the newly created domain does not collide with another domain within the same chromosome. This is calculated on the basis of a condition described by the Eq (11).

$$\sqrt{\left({\left(xyz[0][n_{b}]-tab[1][i]\right)}^{2}+{\left(xyz[1][n_{b}]-tab[2][i]\right)}^{2}+{\left(xyz[2][n_{b}]-tab[3][i]\right)}^{2}\right)}<(2\cdot r_{b}-2\cdot \varepsilon_{1})$$ (11)

The same function is also used to check whether there is not a collision with another domain within another chromosome. The only difference is in *ε* parameter–here *ε*_2_ (which is greater than *ε*_1_) is used. This condition is described by the Eq (12).

$$\sqrt{\left({\left(xyz[0][n_{b}]-tab[1][i]\right)}^{2}+{\left(xyz[1][n_{b}]-tab[2][i]\right)}^{2}+{\left(xyz[2][n_{b}]-tab[3][i]\right)}^{2}\right)}<(2\cdot r_{b}-2\cdot \varepsilon_{2})$$ (12)

Both conditions (11) and (12) have to be met to accept drawn coordinates. Otherwise, the function runs again to draw new set of domain coordinates.

**bead_generate_wrap()** This function is used to restart the program when it cannot find the solution (when it is impossible to add a new domain to the previously created model components). The function counts the number of failures in the process of generating a new domain. When the count reaches the value declared by restart_after variable, the process of model creation is restarted. In the case of restart, the previously generated part of the model is not saved, and the process of generating chromosomes starts from the beginning. The number of restarts does not affect the declared number of models to generate.

### Benchmarks

In order to assess the efficiency of the modeller and the average time needed to generate a single nucleus simulation several benchmarks were run. The tests were performed on a computer with the following parameters: processor i7-950, 3.06 GHz; 20 GB RAM; HDD 7200 rpm 32 MB cache; OS: Win7 Pro 64bit.

#### Configuration parameters used in benchmarking

We tested three benchmark data sets that reflected various types of genome organisation. The first set (Set 1) corresponds to the karyotype of *B*. *distachyon* that comprises five pairs of chromosomes of the size ranging from 28 Mbp to 75 Mbp and length varying from 3.5–7 μm in their condensed state [8, 18]. The other two sets used for benchmarking were roughly based on the genomes of human (Set 2) and biofuel plant *Miscanthus sinensis* (Set 3). Both genomes are characterised by numerous chromosome pairs but differ with regards to the condensed chromosomes’ length. The detailed information about the input data for modelling the nuclei of these species are presented in Table 1. The two additional modeller script files that contain the configuration parameters for set 2 and set 3 can be found in the directory “Config_sets” in Github in the project repository.

**Table 1:** Input data used in benchmarking.

Set	No. of chromosome pairs	Size of chromosomes [Mb]	Length of condensed chromosomes [μm]
1	5	28–75	3.5–7.0
2^a^	23	57–249	1.5–10.9
3^b^	19	87–244	1.4–3.9

^a^ Size data estimated for a male karyotype [19] and calculated using the normalised chromosome lengths and the total length of the karyotype [20].

^b^ Data on chromosome length obtained thanks to Ms Alicja Kotarska (data not published). The size of the chromosomes calculated on the basis of normalised length of condensed chromosomes and total karyotype length [21].

#### Benchmarking procedure and results

The modeller was run to obtain ten models for each configuration set (Table 2). The parameters measured for the purpose of benchmarking were:

- Total computing time (TCT)–time necessary to generate 10 models,
- Number of restarts (NR)–restart by invoking wrap_bead() function takes place in a situations, when a model does not converge after a given number of iterations. The number of iterations before the modelling is restarted can be set through the parameter restart_after. Increasing this parameter will increase the number of iterations and result in longer time before restarting.
- Average time (AT)–average time that is necessary to successfully create one model.

The total computing time for the tested configuration sets varied from 47 min in the case of set 1 to nearly 3 hours in the case of set 3 (Table 2). The differences in TCT seemed to reflect the differing number of chromosomes used in each configuration set (Table 1). For each set, the success rate defined as the percentage of models that have been completed successfully was calculated. In the case of set 1, the procedure had to be run 18 times in order to obtain 10 models (56% success rate), whereas for the configuration sets 2 and 3, the modeller had to be run 11 (90% success rate) and 13 (77% success rate) times, respectively. The relatively low success rate in the case of configuration set 1 was compensated by short computing time, but assessing the relationship between the features of the configuration set and success rate would require further, more extensive analysis.

**Table 2:** Total computing time (TCT), number of restarts (NR) and average time of creating one model (AT) for all configuration sets used in benchmarking.

Configuration	TCT [min]	NR^a^	AT [min]
set 1	47.77	8	0.82
set 2	120.78	1	8.3
set 3	165.22	3	11.1

^a^ The restart_after parameter value set to 500 000.

**Fig 7:**
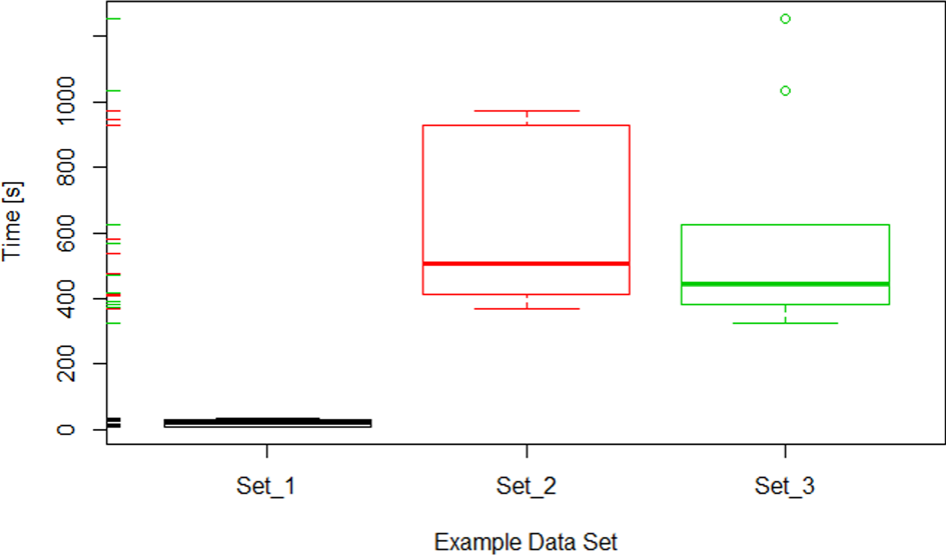
Boxplot showing the range of the computing time for all configuration sets used in benchmarking. The modeller was run to successfully generate ten models for each configuration set. The bolded lines inside each of the boxes indicate the average computing time.

The computing time needed to generate each of the ten models was almost the same in the case of configuration set 1 (Fig 7). For the set 2, the computing times were the most diversified among the tested configuration sets, but had no outliers. In the case of third configuration set, in general, the computing times were less diversified than for the set 2, but two outliers appeared. These results show that the modeller is capable to simulate CTs distribution for different species in a reasonable amount of time and that the average time of a single simulation depends strongly on the input parameters for modelling a given species.

## Results

Our model has been created in order to complement the experimental data on CT distribution in *B*. *distachyon* interphase nuclei. The proposed model enables adjusting species specific parameters so it can be used for simulation of CTs in any species. In order to assess the model usability, the visualisations of simulated nuclei were compared to the photomicrographs that documented experimental data.

### Experimental procedure

Young roots collected from the 3-day old *B*. *distachyon* seedlings were used for experimental analysis of chromosome territory distribution. The seeds of *B*. *distachyon* reference line Bd21 were obtained from the USDA-ARS collection. The method of isolated nuclei preparation was adopted from [22]. Sets of Bacterial Artificial Chromosome (BAC) clones originating from two genomic *B*. *distachyon* libraries [8, 23] were used as chromosome-specific probes for painting chromosome territories. DNA from each BAC clone was isolated using standard alkaline extraction as described by [24], pooled and labelled with tetramethyl-rhodamine-5-dUTP or digoxygenin-11-dUTP. BAC DNA probes were labelled by nick translation using a commercial kit (Roche). Both labelling and FISH were carried out using the protocols published by [9] and [25]. All images of the interphase nuclei were acquired using an Olympus FV1000 confocal system equipped with a 60x PlanApo objective. Image stacks were acquired by traversing from top to bottom in 200 nm steps. Image processing (including Z-stacks rendering in 3-D) and construction of 3-D models of nuclei with the use of, Contour surface” wizard were executed with Imaris software (Bitplane).

### Experimental results

**Fig 8:**
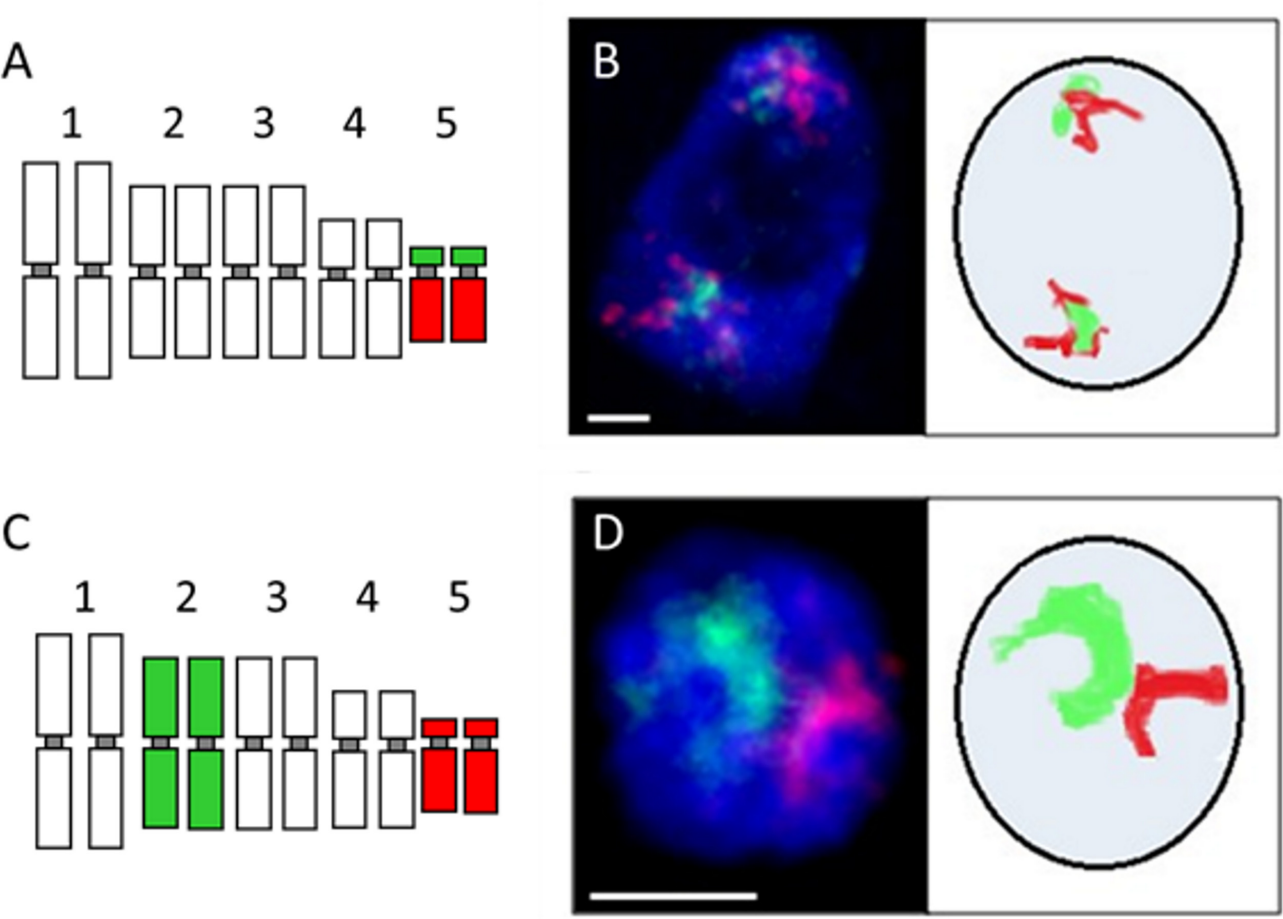
Association of homologous and heterologous chromosome territories in *B*. *distachyon* interphase nuclei of roots. A–an idiogram showing the labelling of Bd5 chromosome (top arm–green, bottom arm–red; each nucleus contains two sets of chromosomes). B–the nucleus with complete separation of Bd5 chromosome arm territories revealed by FISH. Nucleolus is visible as dark blue spherical gap inside the nucleus. C–an idiogram showing the labelling of two heterologous chromosomes (Bd2 –green, Bd5 –red), without discrimination between chromosome arms. D–the nucleus with association of Bd2 and Bd5 chromosome territories revealed by FISH. Photomicrographs in B and D are supplied with diagrams representing the distribution of CTs. Scale bars: 2 μm.

Different experimental strategies were adopted for the analysis of homo- or heterologous chromosome territories (CTs). In the case of mapping of a homologous chromosome pair, the top and bottom arm were labelled with two different fluorochromes (Fig 8A and 8B). Such approach allows to assess the occurrence and frequency of somatic homologue association. When two pairs of heterologous chromosomes were mapped simultaneously, each pair was labelled with different fluorochrome without discriminating between the chromosome arms (Fig 8C and 8D). For the analysis of the association of homologous chromosomes, 100–200 nuclei were examined, depending on the chromosome pair. The simultaneous mapping of two chromosome pairs proved to be more technically challenging. In this case, only ~20 nuclei were suitable for analysis. FISH revealed clearly visible, distinct CTs in both types of experiments (Fig 8). No overlapping of territories was observed. The distribution of homologous chromosome CTs varied from complete separation through the single-arm-only association to whole-length association. The CTs were considered to be associated if the distance between their borders was less than 500 nm. The size of a territory usually reflected the length of the respective chromosome or chromosome arm. In few cases two chromosomes/chromosome arms that differed significantly in their length occupied areas of comparable size, which can be probably explained by the difference in chromatin condensation level.

### Input data for simulating *B*. *distachyon* CTs

The karyotype of *B*. *distachyon*, modelled in this study, consists of five pairs of homologous chromosomes. During mitotic metaphase, when chromosomes are the most condensed, their length ranges from 3.5 μm (chromosome Bd5) to about 7 μm (chromosome Bd1) [18]. In our simulation the assumed diameter of chromatin domains equals 500 nm, thus in the model the condensed chromosomes are represented as chains of beads ranging in length from 7 to 14 beads. The morphology of the chromosomes is diverse since the chromosomes Bd1 –Bd3 are metacentric (the top and bottom arms of each chromosomes are of similar length) while chromosomes 4 and 5 are acrocentric (the top arms are much shorter than the bottom arms). The distance of centromeres from the chromosome ends is also taken into account in our example. According to the data obtained by genome sequencing, the DNA content of chromosomes Bd1 –Bd5 equals 75, 59, 60, 48 and 28 Mbp, respectively [8]. These numbers equal the final numbers of 1 Mbp chromatin domains constituting the decondensed chromosomes. The size of nucleus and nucleolus was based on the experimental data (Robaszkiewicz, data not published).

### Computational results and comparison with experimental data

**Fig 9:**
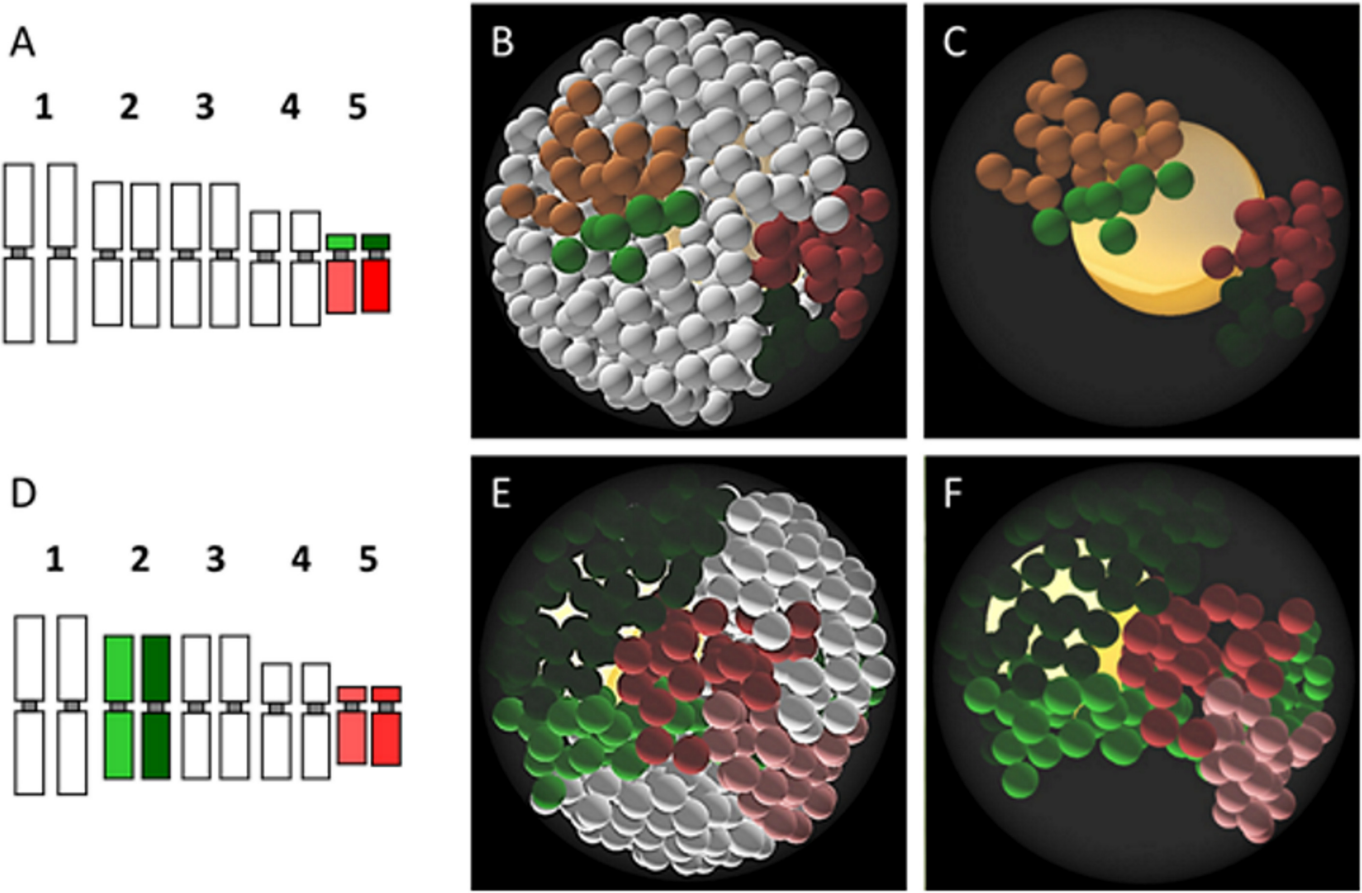
Exemplary visualisation of nuclei modelled by ChroTeMo and visualised by ChroTeVi. A–an idiogram showing the colouring of Bd5 chromosome (top arm–green, bottom arm–red; a nucleus contains two sets of chromosomes). B–the nucleus with complete separation of Bd5 chromosome arm territories. Chromosomes other than Bd5 are coloured white. C–the same nucleus as B. Chromosomes other than Bd5 are transparent. Nucleolus is visible as a yellow sphere inside the nucleus. D–an idiogram showing the colouring of two heterologous chromosomes (Bd2 –green, Bd5 –red), without discrimination between chromosome arms. E–the nucleus with association of Bd2 and Bd5 chromosome territories. Chromosomes other than Bd2 and Bd5 are coloured white. F–the same nucleus as E. Chromosomes other than Bd2 and Bd5 are transparent. Nucleolus is visible as a yellow sphere inside the nucleus.

**Fig 10:**
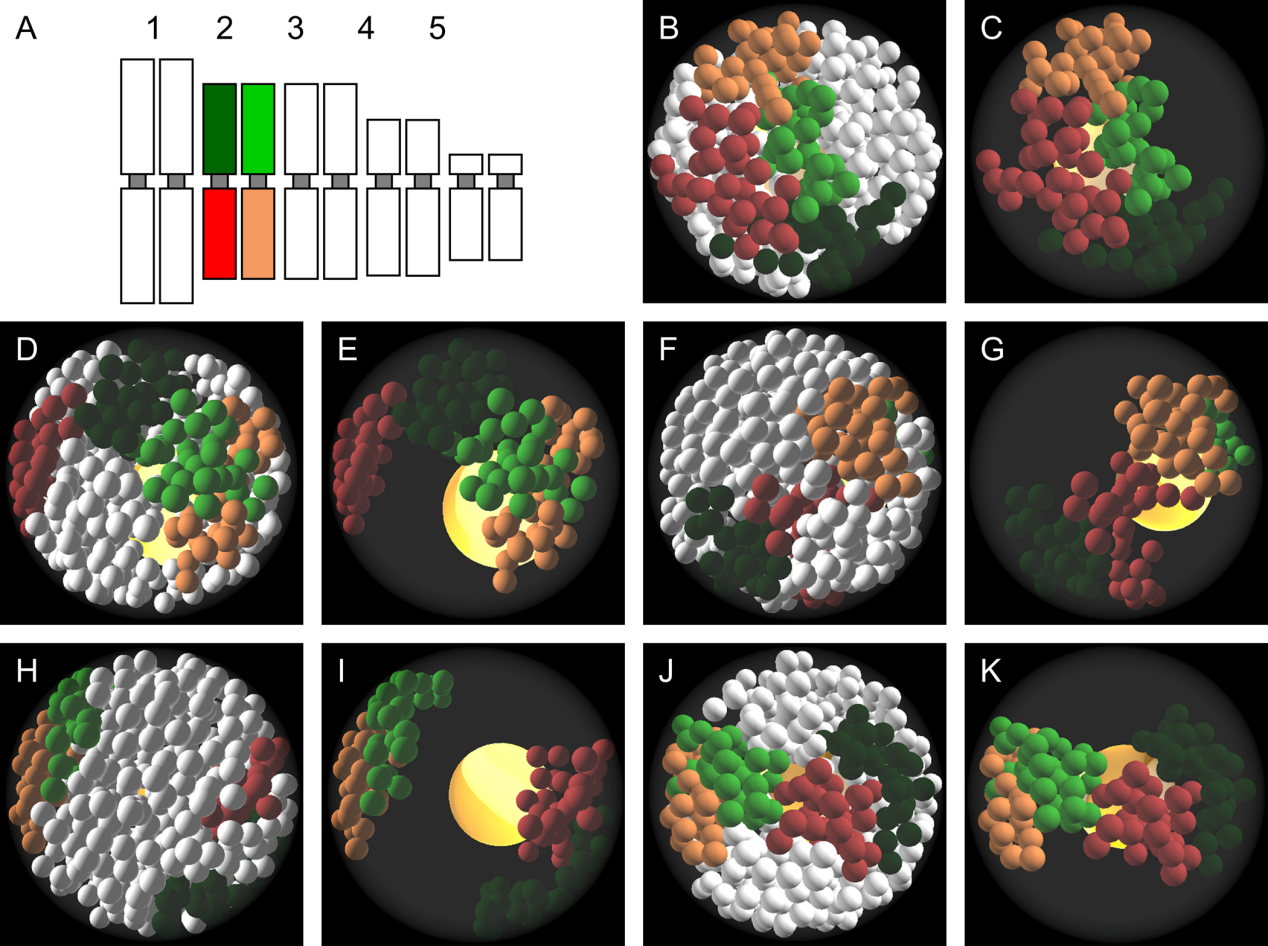
Types of homologous CT distribution patterns modelled by ChroTeMo and visualised by ChroTeVi. A–an idiogram showing the colouring of Bd2 chromosome (top arm–green, bottom arm–red). B–the nucleus with complete association of Bd2 chromosome arm territories. Chromosomes other than Bd2 are coloured white. C–the same nucleus as B. Chromosomes other than Bd2 are transparent. D–the nucleus with the association of Bd2 top arm territories. Chromosomes other than Bd2 are coloured white. E–the same nucleus as D. Chromosomes other than Bd2 are transparent. F–the nucleus with the association of Bd2 bottom arm territories. Chromosomes other than Bd2 are coloured white. G–the same nucleus as F. Chromosomes other than Bd2 are transparent. H–the nucleus with complete separation of Bd2 chromosome arm territories. Chromosomes other than Bd2 are coloured white. I–the same nucleus as G. Chromosomes other than Bd2 are transparent. J–the nucleus with association between the top arm of one Bd2 homologue and the bottom arm of another homologue. Chromosomes other than Bd2 are coloured white. K–the same nucleus as J. Chromosomes other than Bd2 are transparent. Nucleolus is visible as a yellow sphere inside the nucleus.

In order to assess how accurately our model corresponds to the experimentally obtained data, the simulation was run 115 times and the positions and associations of CTs simulated by ChroTeMo were analysed. ChroTeVi was used to colour and visualise individual homologous or heterologous chromosomes. The colouring of chromosomes corresponded to the fluorescent labels used in the experimental procedure. The modelled nucleus was filled fully and uniformly with the generated chromatin. The simulation created distinct, well defined territories for every chromosome (Fig 9). The neighbouring CTs did not overlap, although an occasional intermingling of the simulated chromatin fibres was observed at the border between two adjacent territories. All these features indicate that the behaviour of the real CTs is well mimicked by ChroTeMo. The occurrence of various types of CT arrangement was assessed. As in the case of the experimental results, two adjacent CTs were classified as associated when the distance between their boundaries was less than 500 nm. The analysis of simulation results showed that there were five distinct types of homologous CT distribution patterns (Fig 10). Four of them (complete separation and three types of association: top arm:top arm, bottom arm:bottom arm and along both arms) were found also in the nuclei of *B*. *distachyon* after FISH. The association between the top arm of one homologue and the bottom arm of another homologue was observed in the modelled nuclei, but has never been seen in the experimentally obtained images of the nuclei (Fig 10J). The frequency of such configuration varied from 6.1% to 10.4% for particular simulated chromosome pairs. The Pearson’s Chi-squared test of goodness of fit showed that the differences in the frequency of particular CT configurations between the theoretical and experimental data could not arose by chance (χ2 = 142.4; P <0.001). Since the proposed computational model is fully probabilistic, this difference can indicate that the arrangement of CTs in the ‘real’ nuclei is not random, but is influenced by various factors. In the case of heterologous chromosome pairs, the range of observed reciprocal arrangement of CTs was much broader, which is in agreement with the experimental data. The implementation of ChroTeVi in VPython enabled precise analysis of CTs distribution due to the possibility of rotating the scene and zoom it in and out. The option to view only the selected chromosome pairs, with the rest of the chromatin being transparent, would be particularly helpful in a detailed analysis of the CT position, shape, and neighbourhood within the nucleus interior.

The quantitative assessment of the distances between homo- and heterologous chromosomes should be complemented with an analysis of the frequencies of particular distribution patterns, their comparison between simulated and experimental data, and the calculation of the statistical significance of the observed differences. All these data together would provide information about nucleus architecture that would serve as a reference point in future studies on chromatin organisation dynamics. According to many reports, the localisation of CTs in the nuclei of human and various animals is not entirely random but depends on chromosome gene density, chromosome size, and the ratio between these two parameters [26–30]. Moreover, it has been shown that the distances between homologous chromosomes are larger than the distances between the heterologues [28, 31]. In a model plant *A*. *thaliana* and its relative *A*. *lyrata*, the NOR-bearing chromosomes display the tendency to associate more often than expected if their position was random [7, 32].

## Conclusions and Future Work

The Chromosome Territory Modeller and Viewer have been created in order to simulate the chromosome decondensation processes after nucleus division and to analyse the distribution pattern of the resulting chromosome territories. The parameters of the model can be adjusted to simulate CTs in any other species. The comparison between simulated and experimental data indicated that the proposed prototype model reflects well the organisation of chromatin in the real interphase nuclei. However, some improvements of the model and extensions of its applications are planned for future work. The demonstration model allows for the colouring of a maximum of two chromosome pairs using four different colours. Future work will focus on increasing the number of simulated chromosomes which can be simultaneously coloured and visualised. In this prototype, nucleus and nucleolus are represented as spheres. Within the set of primitives offered by VPython, an ellipse can also be defined so that it is possible to enhance the simulation in the future by including different shapes of the nucleus, which is the case in some differentiated cells. This will require developing additional functions for computations that will enable independent changes in every dimension. Moreover, in order to better assess the spatial relationship between CTs, a dedicated analysis software, which will allow for precise measurements of the distances between the territories, their shape, and span, would be a very useful and desirable addition to ChroTeMo and ChroTeVi.

## Supporting Information

S1 Source Code and Exemplary Models (7Z)

S1 User Manual (7Z)
